# The association between urinary BPA concentrations and urinary incontinence in women

**DOI:** 10.1038/s41598-025-99079-5

**Published:** 2025-05-12

**Authors:** Qian Lyu, Yu Nie, Jianyong Gao, Dong Wang

**Affiliations:** 1 Robot Minimally Invasive Center, Sichuan Academy of Medical Sciences & Sichuan Provincial People’s Hospital, Chengdu, 610072 China; 2 Urology Department, Panzhihua Central Hospital, Panzhihua, 617000 China

**Keywords:** BPA, Urinary incontinence (UI), Urge urinary incontinence (UUI), Female, NHANES, Environmental sciences, Natural hazards, Medical research, Risk factors, Urology

## Abstract

Urinary incontinence (UI) significantly impacts the quality of life and psychological well-being of female patients. Although emerging evidence suggests potential links between endocrine-disrupting chemicals and pelvic floor disorders, previous studies on the association between bisphenol A (BPA) exposure and UI in women have yielded inconsistent results. This study aimed to examine this potential association using data from the 2015–2016 National Health and Nutrition Examination Survey (NHANES) (*n* = 467). Through multiple logistic regression analysis with three adjustment models: Model 1 (crude), Model 2 (adjusted for socio-demographic factors: age, race/ethnicity, education, marital status, and poverty ratio), and Model 3 (further adjusted for BMI, hypertension, diabetes, alcohol/smoking status, and delivery history), we assessed BPA exposure categorized into quartiles. No significant associations were observed between BPA exposure and either stress urinary incontinence (SUI) or mixed urinary incontinence (MUI) across all models (*P* > 0.05). However, participants in the highest BPA quartile (> 7.6 ng/mg creatinine) exhibited a significantly increased risk of urge urinary incontinence (UUI) in Model 1 (OR = 2.01, 95% CI [1.12–3.63]), Model 2 (OR = 2.04, 95% CI [1.08–3.85]), and Model 3 (OR = 2.48, 95% CI [1.18–5.20]). This study has several limitations, including its cross-sectional design, reliance on self-reported UI outcomes, single measurement of urinary BPA, and potential residual confounding from unmeasured factors. While these findings suggest that environmental BPA exposure may contribute to UUI risk in women, future longitudinal studies with repeated biomarker measurements and objective UI assessments are needed to confirm these observations and explore potential mechanisms. If validated, reducing BPA exposure through public health interventions could emerge as a novel preventive strategy for UUI.

## Introduction

Urinary incontinence (UI), defined as the involuntary loss of urine, is traditionally classified into three subtypes: stress urinary incontinence (SUI), characterized by leakage during physical exertion, urge urinary incontinence (UUI), characterized by leakage preceded by a sudden and compelling urge to void, and mixed urinary incontinence (MUI), which involves symptoms of both SUI and UUI. This condition imposes substantial physical, psychological, and economic burdens, with nearly 50% of women experiencing UI during their lifetime and annual U.S. healthcare costs exceeding $76 billion^1^. The integrity of pelvic floor musculature and coordinated bladder-urethral sphincter interactions are crucial for maintaining continence. Disruption of this neuromuscular equilibrium—whether due to obstetric trauma, neurogenic bladder dysfunction, or age-related atrophy of the detrusor and urethral sphincter muscles—substantially increases the risk of urinary incontinence (UI)^2^. Although established risk factors like parity and obesity are primarily associated with SUI, the etiology of urgency urinary incontinence remains poorly understood, particularly concerning potential environmental contributors^3^.

Millions of tons of plastic products are produced worldwide every year, despite the regulatory measures taken. A previous study^4^ reported that plastic products were found in human stool and predicted that more than 50% of the world’s population would be affected, suggesting that people have been inevitably exposed to plastic products and their harmful components. Bisphenol A (BPA), a high-production-volume endocrine-disrupting chemical (EDC) with global production exceeding 10.2 million tons, has been widely implicated in multiorgan toxicity. Accumulating evidence from epidemiological and toxicological studies^5–8^ demonstrates that BPA exposure adversely impacts cardiovascular homeostasis, reproductive function (e.g., impaired spermatogenesis and fetal growth restriction), mammary gland development, and neuroendocrine regulation. Furthermore, BPA is associated with a spectrum of chronic pathologies, including obesity, anxiety/depression disorders, and hormone-dependent malignancies such as breast and prostate cancers. Notably, due to estrogen-mimicking structure, BPA interacts with both classical and non-classical estrogen receptors, triggering diverse molecular pathways that may contribute to bladder dysfunction^9^. First, as an estrogen receptor agonist, BPA may disrupt pelvic floor musculature estrogen signaling crucial for maintaining urethral closure pressure. Second, neurotoxic effects including altered VIP (vasoactive intestinal peptide) expression in bladder neurons could impair detrusor muscle regulation. Third, oxidative stress generation through mitochondrial dysfunction may damage bladder afferent nerves, potentiating urinary urgency^10–12^. Recent animal studies^13^ further demonstrate that BPA exposure induces detrusor instability and increases bladder mass in mice, mirroring human UUI pathophysiology.

Emerging epidemiological evidence indicated that BPA may contribute to multiple reproductive disorders including polycystic ovary syndrome and endometriosis^14,15^. Research on the potential relationship between BPA and UI remains limited. Utilizing data from the 2015–2016 NHANES, this study aimed to investigate the association between urinary BPA levels and the risk of SUI, UUI, MUI in a nationally representative sample of adult women.

## Materials and methods

### Study population

Data from the 2015—2016 NHANES survey cycle were used to study the association between urinary BPA levels and UI. The NHANES is conducted by the CDC’s National Center and collects data representing the health and nutritional status of the U.S. population. As a cross-sectional survey, it employs standardized interviews, physical examinations, and laboratory tests through a multistage probability sampling design. All participants provided signed informed consent, and the data can be publicly accessed on the NHANES website (https://www.cdc.gov/nchs/nhanes/). The survey was approved by the NCHS Research Ethics Review Board (ERB).

In our study, we established rigorous inclusion and exclusion criteria. The inclusion criteria comprised: (1) Female participants aged ≥ 20 years from the 2015–2016 NHANES cohort; (2) Completed both the *Kidney Conditions* Questionnaire (providing personal interview data on kidney disease, stones, urinary incontinence, and nocturia) and urinary BPA—testing modules.; (3) Availability of complete demographic data (age, race/ethnicity). Initially, 5080 candidates were identified. Subsequently, exclusion criteria were applied: (1) participants were diagnosed with urinary tract infection (UTIs); (2) Missing urinary BPA data; (3) Urinary creatinine concentrations < 30 or > 300 mg/dL, indicating renal dysfunction or dilutional errors^16^; (4) Self-reported history of neurological disorders or pelvic irradiation; (5) Pregnancy or postpartum status (≤ 6 months) to exclude obstetric-related transient UI; (6) Missing age/ethnicity information (*n* = 12). After screening, 467 participants were included. Consistent with NHANES methodology, the study employed a stratified multistage probability sampling design to ensure national representativeness. All analyses incorporated survey weights for generalizability to U.S. women ≥ 20 years. Post-weighting, the effective sample size represented approximately million women. Urinary BPA levels were creatinine-corrected to account for dilution effects, thereby minimizing analytical bias^17^. Demographic characteristics stratified by UI status are detailed in Table 1.

**Table 1:** Baseline characteristics of study population by UI subtypes.

	UUI status		SUI status		MUI status	
	No	Yes	No	Yes	No	Yes
Population	352	115	345	122	407	60
Vaginal deliveries	2.6 ± 1.7	2.3 ± 1.4	2.5 ± 1.6	2.6 ± 1.8	2.5 ± 1.7	2.5 ± 1.4
Cesarean deliveries	1.0 ± 0.7	1.0 ± 0.6	1.0 ± 0.8	1.0 ± 0.5	1.0 ± 0.7	0.8 ± 0.5
Age (years) (%)						
20.0-35.0	24.2	28.5	23.6	29.1	24.6	28.7
35.0-47.0	26.7	23.4	29.2	17.1	27.7	12.4
47.0-62.0	20.5	24.6	20.6	23.2	20.4	28.6
63.0-80.0	28.7	23.5	26.7	30.6	27.4	30.3
BMI (kg/m^2^) (%)						
≥30.0	30.2	39.9	31.6	33.3	30.0	49.7
25.0-29.9	35.4	31.6	33.8	37.2	36.0	23.3
18.5-24.9	31.4	26.9	31.4	28.2	30.9	27.1
<18.5	3.0	1.7	3.3	1.3	3.1	0.0
Race/ethnicity (%)						
Mexican American	18.0	12.0	16.6	17.7	17.6	10.3
Other Hispanic	9.4	10.7	8.3	13.7	9.1	14.5
Non-Hispanic White	37.1	25.4	35.7	32.5	35.2	31.6
Non-Hispanic Black	20.5	21.5	20.5	21.1	20.7	21.0
Other race	15.0	30.4	18.9	15.1	17.4	22.6
Education (%)						
Less than high school	21.0	8.9	19.3	17.1	20.0	7.5
High school grad/GED or equivalent	18.5	20.9	15.4	29.5	18.5	23.0
More than high school	60.5	70.2	65.4	53.4	61.5	69.5
Marital status (%)						
Married	47.8	37.3	49.8	34.2	47.1	34.5
Single	45.8	54.5	43.4	59.2	46.4	56.2
Living with a partner	6.4	8.3	6.8	6.7	6.5	9.3
Poverty ratio (%)						
≤1.0	20.6	15.7	19.8	19.3	20.1	16.0
>1.0	79.4	84.3	80.2	80.7	79.9	84.0
Hypertension status (%)						
Yes	22.6	65.0	28.4	37.3	27.0	62.0
No	77.4	35.0	71.6	62.7	73.0	38.0
Diabetes status (%)						
Yes	7.3	14.8	7.4	12.5	7.9	16.5
No	92.7	85.2	92.6	87.5	92.1	83.5
Smoke at least 100 cigarettes in life (%)						
Yes	42.5	55.2	44.6	45.7	44.5	48.6
No	57.5	44.8	55.4	54.3	55.5	51.4
Alcohol (%)						
Yes	1.3	0.9	1.7	100.0	1.4	100.0
No	98.7	99.1	98.3	0.0	98.6	0.0
BPA quartiles(%)						
Lowest quartile	25.5	19.7	26.4	18.7	25.5	15.3
Second quartile	25.1	23.1	24.2	26.3	25.2	20.6
Third quartile	27.7	26.6	24.6	36.2	26.6	35.3
Highest quartile	21.6	30.6	24.9	18.8	22.7	28.7

NHANES 2015-2016; continuous variables as Mean ± SD; categorical variables as %.

### BPA measurements

In the NHANES 2015–2016 cycle, urinary BPA measurements were conducted on a randomly selected one-third subsample of participants. This approach follows a standardized protocol designed to balance logistical constraints while preserving statistical validity and national representativeness. Urinary BPA concentrations were quantified using online solid-phase extraction coupled with high-performance liquid chromatography-tandem mass spectrometry (online SPE-HPLC-MS/MS) using isotope dilution. Using isotopically labeled internal standards, the method achieved detection limits of 0.1–1.7 µg/L in 100 µL of urine; however, this approach has limitations, such as potential biases introduced by analytical variability (https://wwwn.cdc.Gov/nchs/data/nhanes/public/2015/labmethods/EPHPP_I_MET.pdf). To minimize bias from results below the limit of detection (LOD), we included only BPA detected in at least 75% of samples, and concentrations below the LOD were imputed as LOD divided by the square root of two^18^. Finally, BPA concentrations were adjusted for creatinine to account for urine dilution, with values expressed in ng/mg creatinine^19^.

### Measurement of UI

Urinary incontinence subtypes were classified according to the International Continence Society (ICS) standard definitions, and NHANES UI questions aligned with ICS symptom-based diagnosis for epidemiological studies^20,21^. In the *Kidney Conditions* questionnaire, Stress Urinary Incontinence (SUI) was defined by a ‘Yes’ response to urine leakage during physical activities (such as coughing, lifting, or exercise), whereas Urge Urinary Incontinence (UUI) required both affirmation of leakage and an urgent inability to reach a toilet in time during the past 12 months. Participants meeting both criteria were classified as having Mixed Urinary Incontinence (MUI). However, the potential limitations associated with self-reported UI data include recall bias and social desirability bias.

### Statistical analysis

The statistical packages R (The R Foundation; http://r-project.org; v3.4.3) and EmpowerStats (X&Y solutions Inc.; www.empowerstats.com) were used for data analysis. A complex sampling design and survey weights were adjusted to account for the National Health and Nutrition Examination Survey (NHANES) sampling framework, with a two-sided *P* < 0.05 considered statistically significant. To address right-skewed distributions, creatinine-corrected urinary BPA concentrations (ng/mg creatinine) were naturally log-transformed and multiplied by 1000 prior to analysis. The associations between urinary BPA levels and urinary incontinence (UI) subtypes were evaluated by categorizing BPA exposure into weighted quartiles based on the population distribution. Multivariable logistic regression models were used to calculate adjusted odds ratios (ORs) and 95% confidence intervals (CIs). Confounders were selected based on established biological plausibility from previous literature (e.g., age/race as UI modifiers, BMI-metabolic linkages) and empirical criteria where variables altering BPA-UI effect estimates by > 10% were retained. Socio-demographic factors (education, poverty ratio) adjusted for reporting bias and healthcare access disparities, while clinical/behavioral variables (diabetes, smoking) accounted for detrusor dysfunction pathways and obstetric history (vaginal delivery) represented pelvic floor trauma mechanisms. This dual approach ensured methodological rigor by integrating evidence-based covariates and data-driven thresholds to minimize residual confounding^2,22^. Hence, three main models were constructed: Model 1: Unadjusted; Model 2: Adjusted for socio-demographic factors: age (continuous), race/ethnicity (Mexican American, Other Hispanic, Non-Hispanic White, Non-Hispanic Black, Other Race), education (< high school, ≥high school), marital status (married, living with partner, single), and poverty-income ratio (PIR ≤ 1.0 vs. >1.0); Model 3: Further adjusted for clinical/behavioral factors: BMI (continuous), hypertension (yes/no), diabetes (yes/no), smoking status (≥ 100 lifetime cigarettes), alcohol use (yes/no), and vaginal delivery history (yes/no). Participants with missing covariates (*n* = 12, 2.6% of the cohort) were excluded via complete-case analysis, as sensitivity analyses showed no systematic differences between excluded and included participants^23^.

## Results

The weighted distributions of the study population (*n* = 467) characteristics of the total sample are included in Table 1. Mean age (± standard deviation) was comparable across groups: 49.8 ± 18.7 years in the SUI group, 51.0 ± 18.2 years in the UUI group, 48.3 ± 14.4 years in the MUI group, and 50.4 ± 17.3 years in the non-UI group, with no statistically significant intergroup differences observed (one-way ANOVA, *P* > 0.05). Of those 467 women, 115 reported UUI, 122 reported SUI, 60 had MUI, and 170 had no UI. In the UUI group, women aged 20—35 years formed the greatest proportion (28.5%), whereas in the SUI and MUI groups, women aged 63—80 years accounted for the greatest proportion (30.6% and 30.3%, respectively). Patients with UI and a BMI ≥ 25 accounted for 71.5% of the UUI group, 70.5% of the SUI group, and 73% of the MUI group. The mean number of vaginal deliveries (± SD) was 2.3 ± 1.4 in the UUI group, 2.6 ± 1.8 in the SUI group, and 2.5 ± 1.4 in the MUI group. The majority of the participants were non-Hispanic White. Table 2 lists the geometric means and median urinary concentrations of BPA by UI status. Creatinine-adjusted urinary BPA concentrations (mean ± SD) varied across urinary incontinence subtypes. Participants with UUI exhibited significantly higher levels compared to non-UUI individuals (7.3 ± 1.0 vs. 7.0 ± 0.9 ng/mg creatinine; *P* < 0.05). In contrast, SUI and non-SUI groups had identical concentrations (7.1 ± 0.9 ng/mg creatinine; *P* > 0.05). Notably, non-MUI cases had significantly lower BPA levels than MUI cases (7.0 ± 0.9 vs. 7.3 ± 1.0 ng/mg creatinine; *P* = 0.048). The distribution of BPA quartiles was divided as follows: <6.5 ng/mg creatinine (Q1), 6.6—7.1 ng/mg creatinine (Q2), 7.2—7.6 ng/mg (Q3), and > 7.6 ng/mg creatinine (Q4). For Q1, Q2, Q3, and Q4 of BPA, 19.7%, 23.1%, 26.6%, and 30.6% of the females reported a history of UUI; 18.7%, 26.3%, 36.2%, and 18.8% reported SUI; and 15.3%, 20.6%, 35.3%, and 28.7% reported MUI, respectively.

**Table 2:** Creatinine-corrected BPA concentrations by urinary incontinence (UI) subtypes.

UI subtype	Yes/no	Mean ± SE (ng/mg crt)	Median [IQR] (ng/mg crt)	*P* value
UUI	No	7.0 ± 0.9	7.0 [6.4–7.5]	*P*<0.05
*(n=115)*	Yes	7.3 ± 1.0	7.2 [6.6–7.9]	
SUI	No	7.1 ± 0.9	7.0 [6.5–7.6]	*P*>0.05
*(n=122)*	Yes	7.1 ± 0.9	7.1 [6.4–7.6]	
MUI	No	7.0 ± 0.9	7.0 [6.5–7.6]	*P*<0.05
*(n=60)*	Yes	7.3 ± 1.0	7.2 [6.6–8.0]	

Values presented as Mean ± SE or Median [IQR]; ng/mg crt represented ng/mg creatinine; NHANES 2015-2016.

**Fig. 1:**
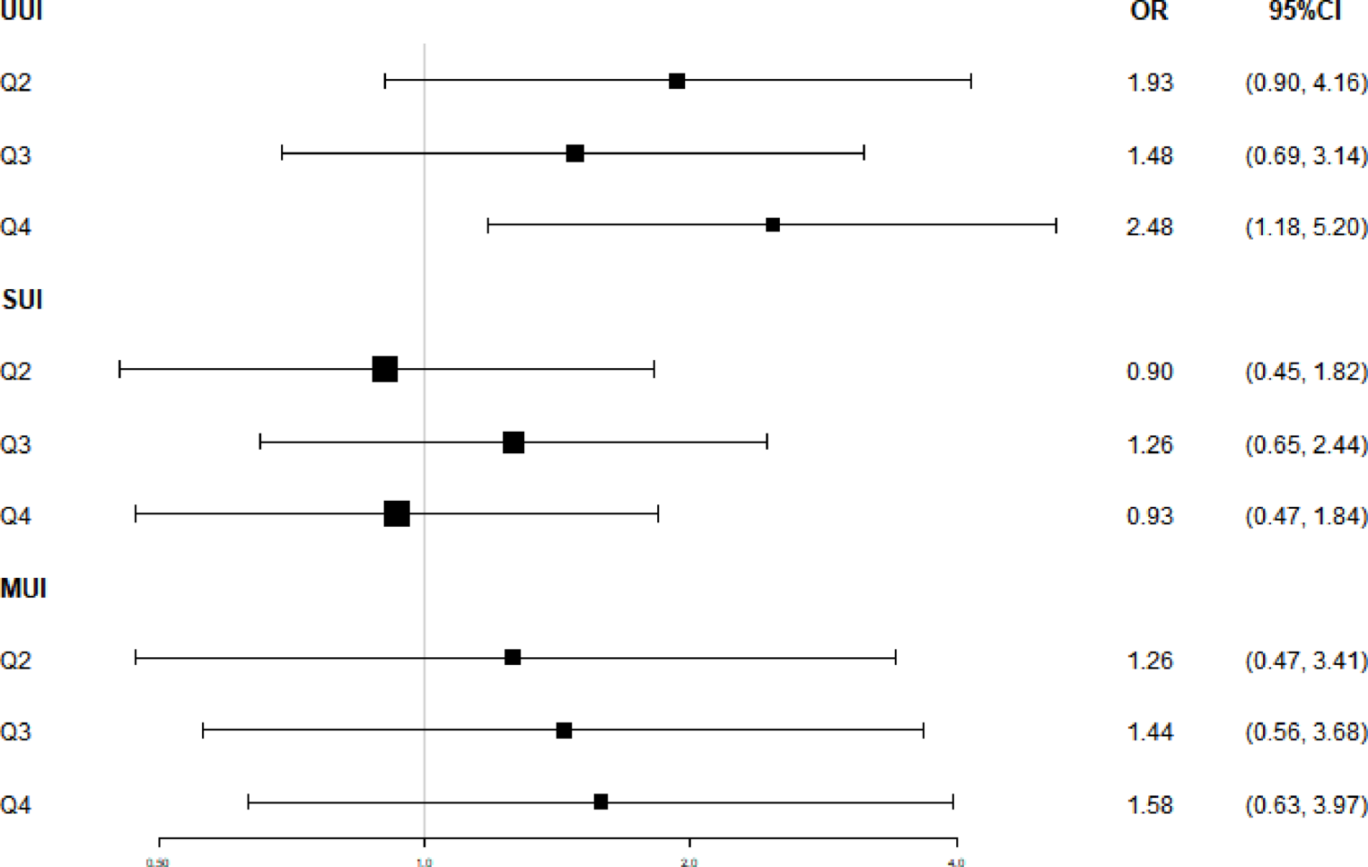
Adjusted ORs and CIs of UI stratified by type and concentration of creatinine-corrected BPA; NHANES 2015–2016.

Table 3; Fig. 1 present the results of the multivariable logistic regression analysis. No significant associations between BPA exposure and UI were observed for SUI or MUI in Model 1 (unadjusted, *P* > 0.05). These results remained consistent in Models 2 and 3 after further adjustment for covariates (*P* > 0.05). In the UUI group, compared with those in the lowest quartile, the odds ratios (ORs) for the second quartile and the third quartile were not significantly different, regardless of covariate adjustment. However, the ORs of the highest quartile were significantly greater than those of the lowest quartile in Model 1 (OR = 2.01, 95%CI [1.12, 3.63]), and the results remained significant after adjusting for variables in Model 2 (OR = 2.04, 95%CI [1.08, 1.85]) and Model 3 (OR = 2.48, 95%CI [1.18, 5.20]), which indicates that the highest quartile of BPA was significantly associated with UUI.

**Table 3:** Association between BPA quartiles and urinary incontinence subtypes.

	Model 1	*P* value	Model 2	*P* value	Model 3	*P* value
UUI						
Q1: <6.5 ng/mg crt	Referent	–	Referent	–	Referent	–
Q2: 6.6-7.1 ng/mg crt	1.03 (0.55, 1.95)	*0.92*	1.23 (0.62, 2.41)	0.09	1.93 (0.90, 4.16)	0.32
Q3: 7.2-7.6 ng/mg crt	1.14(0.61, 2.12)	*0.68*	1.09 (0.56, 2.12)	0.56	1.48 (0.69, 3.14)	0.80
Q4: >7.7 ng/mg crt	2.01(1.12, 3.63)	*0.02*	2.04 (1.08, 3.85)	0.03	2.48 (1.18, 5.20)	0.02
p trend	0.02		0.04		0.04	
SUI						
Q1: <6.5 ng/mg crt	Referent	–	Referent	–	Referent	–
Q2: 6.6-7.1 ng/mg crt	0.79 (0.43, 1.43)	0.44	0.77 (0.40, 1.48)	0.44	0.90 (0.45, 1.82)	0.77
Q3: 7.2-7.6 ng/mg crt	1.06 (0.60, 1.88)	0.84	1.18 (0.64, 2.19)	0.60	1.26 (0.65, 2.44)	0.49
Q4: >7.7 ng/mg crt	0.92(0.51, 1.64)	0.78	0.89 (0.47, 1.67)	0.72	0.93 (0.47, 1.84)	0.84
p trend	0.97		0.93		0.91	
MUI						
Q1: <6.5 ng/mg crt	Referent	–	Referent	–	Referent	–
Q2: 6.6-7.1 ng/mg crt	0.71 (0.30, 1.67)	0.43	0.85 (0.34, 2.10)	0.73	1.26 (0.47, 3.41)	0.65
Q3: 7.2-7.6 ng/mg crt	1.14 (0.53, 2.46)	0.74	1.23 (0.53, 2.81)	0.63	1.44 (0.56, 3.68)	0.45
Q4: >7.7 ng/mg crt	1.52 (0.73, 3.17)	0.27	1.58 (0.70, 3.55)	0.27	1.58 (0.63, 3.97)	0.33
p trend	0.15		0.17		0.31	

Odds ratios [95% CI] across adjustment models; ng/mg crt represented ng/mg creatinine; NHANES 2015-2016.

## Discussion

In our study, the associations between BPA exposure and different types of UI were assessed in a nationally representative cross-sectional study. Our results indicated that women with UUI had significantly higher urinary BPA levels compared with the general population. The highest BPA quartile (> 7.6 ng/mg creatinine) was significantly associated with UUI (OR = 2.48, 95%CI [1.18–5.20]), suggesting a dose-dependent relationship. Moreover, no associations were detected between BPA exposure and SUI or MUI. Studies on this topic remain scarce, and findings are conflicting due to the complex nature of BPA exposure and UI status. Our findings suggest that reducing environmental BPA exposure could serve as a novel preventive strategy for UUI in high-risk populations, such as women with obesity or metabolic disorders. Although causality remains unproven due to the cross-sectional design, these findings underscore the need for clinicians to integrate environmental risk assessments into UI management, including providing patient education on minimizing BPA exposure.

UI is the involuntary loss of urine, where SUI is defined as urine leakage accompanied by a sudden urgency to void, with patients often experiencing sensations that are difficult to control. UUI is characterized by a poor ability to inhibit detrusor muscle contraction, though its risk factors remain unclear^2^. As a widely recognized endocrine-disrupting chemical, BPA can enter the body via multiple ways, including through dermal exposure, ingestion, and inhalation. This compound has been reported to accumulate in the body and poses a long-term threat to health^24^. BPA may impair steroid signaling, thereby interfering with various physiological functions, including development, reproduction and neurological processes^9^.

The observed association between BPA exposure and UUI risk may be mediated through three biological pathways: neurogenic, myogenic, and epigenetic mechanisms. Experimental evidence reveals that BPA exerts significant neurotoxic effects, including reduced synaptic plasticity, suppressed neurogenesis, and induced autophagy/apoptosis, collectively contributing to neuronal dysfunction. Notably, these neurotoxic effects exhibit transgenerational persistence, irrespective of exposure duration^25,26^. Furthermore, BPA promotes reactive oxygen species (ROS) generation in bladder neurons, thereby impairing mitochondrial function and synaptic plasticity. Chronic oxidative stress damages inhibitory GABAergic interneurons in the sacral spinal cord, compromising supraspinal inhibition of bladder contractions^27^. Regarding myogenic mechanisms, emerging evidence highlights BPA’s impact on bladder smooth muscle dynamics. Makowska et al.^11^ demonstrated that varying BPA concentrations reconfigure the distribution of vasoactive intestinal peptide (VIP)-positive neural structures in the porcine bladder trigone. VIP — a critical neuromodulator for bladder muscle activity and blood flow — shows upregulated expression in neurons and fibers following BPA exposure. This hyperinnervation potentiates bladder afferents (C-fibers), amplifying urgency signals to the pontine micturition center. Supporting this, Nguyen et al.^13^ administered BPA via oral gavage to 6–8-week-old mice for two months, observing detrusor muscle instability and increased bladder mass. Concurrently, Taylor et al.^28^ reported perinatal BPA exposure in mice led to urinary flow alterations and bladder hypertrophy, further implicating VIP-mediated afferent sensitization. Additionally, BPA enhances vascular smooth muscle cell proliferation and upregulates angiotensin II expression, which may cross-activate bladder AT1 receptors to exacerbate detrusor contractility^29^. As an endocrine disruptor, BPA interferes with estrogen receptor signaling and other hormonal pathways essential for bladder urothelial integrity and smooth muscle homeostatic maintenance. Such hormonal imbalances may induce bladder hypersensitivity and urinary urgency — pathognomonic features of UUI^30^. Epigenetically, BPA exposure induces DNA methylation and histone acetylation modifications in genes regulating bladder function and neuronal activity^31^. These epigenetic alterations likely dysregulate the expression of bladder control genes, predisposing individuals to UUI pathogenesis.

While current mechanistic understanding of BPA’s role in UI primarily derives from animal models, human epidemiological evidence directly linking BPA exposure to UI subtypes remains limited. Our study addresses this knowledge gap by establishing the first population-based evidence of a dose-dependent association between BPA exposure and urge urinary incontinence (UUI) among women. Although cross-sectional data limit causal inference, our findings—experimentally corroborated by preclinical models—emphasize the necessity for longitudinal human studies to elucidate BPA’s role in UUI pathogenesis.

Our study has several limitations that warrant cautious interpretation of the findings. First, the cross-sectional design limits causal inference between BPA exposure and UI onset due to temporal ambiguity. Potential reverse causation (e.g. UUI patients modifying dietary habits to avoid BPA—containing products) remains plausible. Second, reliance on self-reported UI outcomes introduces potential recall bias (e.g., underreporting symptoms due to memory lapses) and social desirability bias (e.g., reluctance to disclose incontinence), despite alignment with ICS criteria. Third, urinary BPA was measured only once, reflecting recent exposure rather than chronic patterns critical for UI pathogenesis. Finally, residual confounding persists from unmeasured lifestyle factors (e.g. dietary phytoestrogens, occupational co-exposures to phthalates) and genetic susceptibility (e.g., ESR1 polymorphisms affecting estrogen receptor signaling), which may confound observed associations. Despite these constraints, our study offers novel strengths: it is the first population-based analysis to explore BPA—female UI associations using nationally representative NHANES data (*n* = 467), and the large, weighted sample enhances generalizability.

## Conclusion

The adverse effects on health resulting from constant environmental exposure to EDCs should receive increased attention because of their ubiquitous presence. Our findings suggest that environmental BPA exposure, particularly at higher concentrations (> 7.6 ng/mg creatinine), shows significant association with urge urinary incontinence (UUI) risk in women, demonstrating adjusted odds ratios ranging from 2.01 to 2.48 across models. While causal relationships remain unestablished due to the cross-sectional design and single-time BPA measurement, these results nonetheless highlight notable public health implications. First, targeted reduction of BPA exposure may mitigate the risk of UUI. Second, our findings emphasize the need for longitudinal studies incorporating repeated BPA measurements combined with objective UI assessments to establish causation and investigate underlying mechanisms. Finally, should subsequent validation occur, strategic integration of BPA exposure reduction protocols into clinical guidelines for UUI prevention might complement existing strategies, thereby potentially alleviating the substantial physical, psychological, and economic burdens associated with this condition.

## Data Availability

These data can be publicy accessed from the National Health and Nutritional Examination Survey (NHANES) (https://www.cdc.gov/nchs/nhanes/index.htm).
